# Use of Placental Forceps through the Speculum

**Published:** 1873-04

**Authors:** H. Seaman

**Affiliations:** Millport, N. Y.


ART. IV.—*Use of Placental Forceps through the Speculum.* By H. SEAMAN, M. D., Millport, N. Y.

As the older writers on obstetrics failed to give us much information about the treatment in case a portion of the placenta remained, or was retained in the uterus after abortions, but rather advised trusting such cases to nature, we have been left to grope our way in the dark, aided only by the sense of feeling.

One of our later writers, Churchill on midwifery says: "If the fœtus alone be expelled, we may wait a while, (if no flooding occurs) to see if the uterine efforts will detach the secundines; if not, perhaps we may be able to reach the lower portion of them with our finger, and gradually withdraw them; if this fails, we may frequently succeed with ergot of rye. But there are many cases in which none of these plans will succeed. Are we then to leave the case to nature? We know that after a time the shell of the ovum will putrify, dissolve, and be discharged; but experience too often proves, that this process involves considerable danger; danger of hemorrhage first, and afterwards of uterine phlebitis, and with regard to the danger of phlebitis from absorption of a putrid ovum, it is sufficiently eminent to warrant interference, if we are called early enough." And he further adds, "The French recommend a pair of long thin forceps with which the ovum is to be seized and removed; But against such an instrument there lies this serious objection, that we cannot be certain of not injuring the uterus unless we introduce the fore-finger also." Now it is most evident that if this serious objection could be so easily overcome by simply introducing the fore-finger it would be no objection at all. The finger serves only as a guide to the point of the forcep, and when it reaches the *os*, and we attempt to open the instrument, we are lost as to the situation of one blade. And I trust it may be safely asserted, that no cautious practitioner ever felt perfect indemnity from inquiring the uterus when attempting to use the placental forceps, aided only by the sense of touch. This has been my experience, and it has nearly deterred me from the use of that instrument; and it is to be feared that in bolder hands, it has too often, done irreparable injury.

Is there then any method by which the placental forceps can be used with perfect safety? I think there is; and although the suggestion I am about to make, may not be new to others, it is original with me, and I have failed to find it mentioned in medical journals or books, nor to have heard from the mouths of our brethren. The thought arose in the treatment of the following case, which occurred Sept. 1st, 1872.

Mrs. P., about three months advanced in pregnancy, was taken with labour pains, attended with considerable hemorrhage. I was soon called in, and found the fœtus expelled, with the membranes entire, holding the liquor amnii in the sack, and a small portion of the placenta attached. I removed the clots, and found the bleeding had nearly ceased. I then examined the mouth of the womb and found it contracted to about half an inch, but could not decide definitely, how much of the placenta had been expelled. The pains had ceased. I stayed some time with the patient, and as there was no return of pain, I ordered ergot every four hours, and left, with a promise to call next day, and in the mean time, if any unfavourable symptoms occurred, requested to be called immediately. Sixteen hours after I saw the patient again, and found her comfortable. Each dose of ergot had caused severe pain, but failed to expel anything from the womb. But there had been a little florid discharge from the vagina. The third day the patient was still comfortable.

On the fourth day I was called in haste, and found the woman alarmed from an uneasy sensation which she called a falling down of the womb. This feeling was probably caused by a small portion of placenta lodged in the vagina and an aching pain in the uterus. After removing this portion of placenta, it became apparent that there was still more in the uterus, which could not be brought away with the fingers, and the use of other means seemed necessary. The mention of using forceps, created much alarm in the mind of the patient, and although the introduction was guided by the finger, the moment the instrument was felt in the vagina, she would complain of pain before its point reached the uterus; and such were her fears, that the effort was abandoned.

The thought now occurred, that the mouth of the womb could be reached with forceps through the tube of a speculum; the mention of which at once allayed the fears of the patient.

The speculum was introduced, the *os* brought fairly to view, and cautiously dilated with the forceps, the retained portion of placenta was readily grasped by the instrument through the tube of the speculum, and with a few gentle rotary movements, detached and brought away.

The operation required but little time, causing no fear, and little pain, and in a few days the patient was well.

From my experience I have come to the conclusion that the only safe and reliable method of using the placental forceps, is through the tube of a speculum, and that in this way it can always be used with the utmost safety and assurance in any case where its use may be necessary.

